# Alcohol Induced Depression: Clinical, Biological and Genetic Features

**DOI:** 10.3390/jcm9082668

**Published:** 2020-08-18

**Authors:** Adriana Farré, Judit Tirado, Nino Spataro, María Alías-Ferri, Marta Torrens, Francina Fonseca

**Affiliations:** 1 Institut de Neuropsiquiatria i Addiccions (INAD), Hospital del Mar, 08003 Barcelona, Spain; AFarre@parcdesalutmar.cat (A.F.); mtorrens@parcdesalutmar.cat (M.T.); 2 Grup de Recerca en Addiccions, Institut Hospital del Mar d’Investigacions Mèdiques (IMIM), 08003 Barcelona, Spain; jtirado@imim.es (J.T.); malias@imim.es (M.A.-F.); 3 Psychiatry Department, Universitat Autònoma de Barcelona, Cerdanyola del Valles, 08193 Barcelona, Spain; 4 Genetics Laboratory, UDIAT-Centre Diagnòstic, Parc Taulí Hospital Universitari, Institut d’Investigació i Innovació Parc Taulí I3PT, 08208 Sabadell, Spain; nspataro@tauli.cat

**Keywords:** primary major depression, alcohol use disorder, alcohol induced major depression, biomarkers, comorbidity, clinical characteristics, GWAS

## Abstract

Background: In clinical practice, there is the need to have clinical and biological markers to identify induced depression. The objective was to investigate clinical, biological and genetic differences between Primary Major Depression (Primary MD) and Alcohol Induced MD (AI-MD). Methods: Patients, of both genders, were recruited from psychiatric hospitalisation units. The PRISM instrument was used to establish the diagnoses. Data on socio-demographic/family history, clinical scales for depression, anxiety, personality and stressful life events were recorded. A blood test was performed analysing biochemical parameters and a Genome Wide Association Study (GWAS) to identify genetic markers associated with AI-MD. Results: A total of 80 patients were included (47 Primary MD and 33 AI-MD). The AI-MD group presented more medical comorbidities and less family history of depression. There were differences in traumatic life events, with higher scores in the AI-MD (14.21 ± 11.35 vs. 9.30 ± 7.38; *p* = 0.021). DSM-5 criteria were different between groups with higher prevalence of weight changes and less anhedonia, difficulties in concentration and suicidal thoughts in the AI-MD. None of the genetic variants reached significance beyond multiple testing thresholds; however, some suggestive variants were observed. Conclusions: This study has found clinical and biological features that may help physicians to identify AI-MD and improve its therapeutic approach.

## 1. Introduction

Major Depression (MD) and alcohol use disorders (AUD) are two of the more prevalent mental health disorders in the general population and constitute a major health burden worldwide [1,2]. Clinical [3,4,5,6,7] and epidemiological [8,9,10,11,12] studies show that MD and Alcohol Use Disorder (AUD) frequently co-occur. A systematic review of longitudinal or cross-sectional epidemiological studies found that the presence of either disorder doubled the risk of the second disorder [13], meaning that patients with MD are twice as likely to develop an AUD and vice versa [14].

Diagnosis and treatment of the commonly co-occurring AUD and depressive disorders implies many challenges [15]. Diagnosis is particularly challenging because, as described in other substances with addiction liability, the acute and chronic effects related to alcohol consumption/withdrawal can mimic depressive symptoms. In this sense, MD associated to any SUD has been recognized by both, DSM and ICD classifications for a long time (DSM-IV, IV-TR and DSM-5; ICD-10, ICD-11). The need to differentiate between primary and substance-induced mood disorders has been long-established due to their prevalence and important treatment implications (see systematic reviews published by Schuckit in 2006, Nunes and Levin 2004, Torrens et al., 2005) [16,17,18]. In particular, the differentiation among independent depression or alcohol-induced depression has been extensively studied in terms of characteristics, prognosis, suicide risk and relapse risk among others [11,13,19,20,21,22,23,24,25,26]. Given the available knowledge, it can thus be stated that induced depressive episodes can be as or more serious than primary or independent ones, both in terms of relapse to substance use [3,27] and in the severity of depressive symptomatology [21,28], including risk of suicide [19,22]. This difference may be especially relevant for treatment management [18,23]. In the case of alcohol, each type of depressive episode could be considered as two different diseases since Primary MD patients’ present greater familial risk to develop a primary episode, while this association is not present for the induced episodes [29].

It is has been enough established that depressed patients exhibited elevated levels of C Reactive Protein (CRP) and a significant decrease in their Thyroid-stimulating hormone (TSH) levels, directly related with hypothyroidism [30,31]. Alcohol abuse is a major cause of abnormal liver function and liver enzyme activities are important screening tools for detecting liver disease [32]. Other biomarkers such as cholesterol and triglycerides were previously associated with depression and alcohol use disorder. Although with controversial results, metabolic syndrome, especially lipid dysregulation have been found in primary depression [33,34]; furthermore, alcohol consumption has been related with a tendency towards hypertriglyceridemia [35].

Furthermore, MD and AUD are complex disorders which encompass multiple genetic and environmental factors [30]. Both AUD and MD have substantial genetic contributions with heritability estimates of 50–60% for AUD [31] and 30–40% for MD [32]. Increased familial recurrence risk and heritability have been associated with earlier-onset and recurrent depression [33,34,35] as well as greater depression severity or impairment [36,37].

Common genetic factors that influence the co-occurrence of MD and AUD have been sought in family, twin, and adoption studies [36,37,38,39,40,41,42,43]. GWASs have reported genome-wide significant findings for AUD [44,45] and MD [46,47,48,49,50]. However, no consistent findings have been reported for comorbid AUD and MD [51,52]. Discovering the genetic component of shared liability presents an opportunity to clarify the aetiology of both disorders [51]. Evidence suggests that genetic influences underlying psychiatric and substance use disorders might differ across ancestry groups. In a recent report from Zhou et al. [52], a single genome-wide significant variant was detected, located in the SEMA3A gene. The variant was only common enough to be tested in the African American sample; however, nearby variants in the European American sample that occurred with sufficient frequency to test showed no evidence of association [52].

Given the high prevalence and related negative impact of the comorbidity between AUD and Induced Major Depressions (I-MD), the need to distinguish between co-morbid conditions (i.e., independent psychiatric problems) and conditions where psychiatric symptoms are secondary to substance use has become crucial for clinicians working with substance use disorder patients. As far as we know, there are no studies that characterize Induced Major Depressions (I-MD) from a clinical ad biological perspective to differentiate them from Primary Major Depression (Primary MD). The objective of the present study was to investigate clinical, biological and genetic differences between Primary MD and AI-MD.

## 2. Material and Methods

### 2.1. Design

This is a cross-sectional study comparing two different phenotypes of MD: The Primary MD and the AI-MD.

### 2.2. Participants and Recruitment

From November 2015 to October 2017, a total of 111 patients were assessed for eligibility. Participants were recruited from detoxification, dual diagnosis and acute psychiatric units from the Neuropsychiatry and Addiction Institute of Parc de Salut Mar in Barcelona (PSMAR). Both Primary MD and AI-MD diagnoses were done according to DSM-IV-TR criteria [53]. Inclusion criteria included both genders, aged between 18 and 65 years and of Caucasian origin. Exclusion criteria for both groups were: language barrier or intellectual difficulties that limited the understanding of evaluations, history of pathological conditions or any kind of somatic disorder or disease that the investigator considered unsuitable for the study, other concomitant psychiatric disorder in axis I and any diagnosis of substance use disorder (current or life-time, except nicotine use disorder) (DSM-IV-TR) in the MD group; in the AI-MD group, any other diagnosis of substance use disorder than alcohol use disorder or nicotine use disorder (DSM-IV-TR). Participants from the AI-MD group recruited in the detoxification unit were included in the study at the end of their admission (mean days of admission 13); all of them were under pharmacological treatment of their alcohol abstinence syndrome and also, all participants had punctuations in the Revised Clinical Institute Withdrawal Assessment for Alcohol Scale (CIWA-Ar) below 10 at the inclusion.

### 2.3. Measures

#### 2.3.1. Clinical Assessments

Participants were evaluated using the Spanish version of the Psychiatric Research Interview for Substance and Mental Diseases (PRISM) [54,55] according to “Diagnostic and Statistical Manual of Mental Disorders-4th Edition-Text Revision” (DSM-IV-TR) criteria [53], including a protocol of a family history of depression. In addition, the validated Spanish version of the following instruments were used: severity of depression was assessed using the Spanish validated version of the “Hamilton Depression rating Scale (HAM-D)” [56], the Spanish validated version of the “Beck Depression Inventory (BDI)” [57] and the Spanish validated version of the “Scale for Suicide Ideation (SSI)” [58]. Anxiety severity was evaluated with the Spanish validated version of the “Hamilton Anxiety rating Scale (HAM-A)” [59] and the Spanish validated version of “State-Trait Anxiety Inventory (STAI-R)” [60]. Personality was assessed with the Spanish validated version of the “Temperament and Character Inventory (TCI)” of Cloninger [61]. Traumatic and stressful life events were evaluated with the Spanish validated version of the “Life Stressor Checklist-Revised” (LSC-R) [62].

#### 2.3.2. Blood Samples

A total of 20 mL of blood sample was collected from each participant. From the total, 10 mL was used to conduct a blood test, assessing the levels of C Reactive Protein (CRP), Thyroid-stimulating hormone (TSH), liver function (bilirubin, alanine transaminase (ALT), aspartate transaminase (AST), alkaline phosphatase (ALP), and gamma-glutamyl transpeptidase (GGT)) and lipids (triglycerides and cholesterol). The other 10mL of blood sample was collected to perform the GWAS analysis.

### 2.4. Procedure

The study was approved by the Ethical and Clinical Research Committee of the institution (CEIC number: 2015/6012/I). Written informed consent was obtained from each subject after they received a complete description of the study and had been given the chance to discuss any questions or issues before the start. Study participants were reimbursed with 20 euros for their participation in the study. Participation in this study consisted in one visit of approximately 3 h, where participants were interviewed and blood samples were collected. Genetic samples were adequately stored under professional biobanking procedures until the end of the recruitment period and then prepared for analysis. Blood samples were analysed by the Hospital del Mar (Laboratori de Referència de Catalunya). Genetic samples were adequately stored by UPF-CompOmics under professional biobanking procedures until the end of the recruitment period. Afterwards, biological samples were provided to the Genomics Core Facility service at the National Genotyping Center (CeGen) for sample preparation. Finally, genetic data from CeGen were shared with UPF-CompOmics for analysis.

### 2.5. Data Analysis

#### 2.5.1. Clinical and Blood Tests

Analysis of clinical and blood test data were performed using SPSS Version 23 (IBM SPSS Statistics for Windows (IBM Corp., Armonk, NY, USA). Frequencies, percentages, mean and standard deviations (SD) were calculated. Analysis of the relationship between variables was performed through Chi-Square for dichotomous variables and T-Test (independent samples) for continuous variables. A 5% or lower *p*-value (i.e., <0.05) was considered statistically significant.

#### 2.5.2. Genetic Data

- Genotyping procedure

The protocol used in the processing of this platform is detailed in the user guide “Axiom™ 2.0 Assay Manual Workflow”, available at www.thermofisher.com. In summary, the total genomic DNA was amplified and fragmented up to 25–125 bp. These fragments were purified and re-suspended in the hybridization solution that was transferred to the GeneTitan Instrument to follow its fully automated processing (hybridization in the array plates, staining, washing and scanning). The raw images were automatically processed and the genotypes were obtained by applying the Axiom algorithm, available through the Axiom Analysis Suite software (version, 4.0. Affymetrix, Inc.; Santa Clara, CA, USA, www.thermofisher.com).

- Association analysis

For the association analysis, we used a whole genome association analysis toolset called Plink. We performed 3 different tests separately: (i) basic allelic chi-square; (ii) Fisher’s exact test and (iii) logistic regression to test for differences between the individuals affected by Primary MD and the individuals affected by AI-MD.

Apart from testing each single variant, a covariates analysis was also conducted into the logistic model. A total of 16 different covariates were included in the analysis, all of them related with clinical features considered relevant for depression heritability. These covariates were: gender, age, birth date, race, depression family history, alcohol family history, SUD family history, depression age of onset, severity of depression (HAM-D, BECK and SSI), anxiety scales (HAM-A, STAI-R and STAI-E), number of suicide attempts and live events scale (LSC-R).

## 3. Results

A total of 111 patients were assessed for eligibility. Twenty of them met at least one of the exclusion criteria and eleven refused to participate. A total of 80 participants were included in the study, 47 with Primary MD and 33 with an AI-MD diagnosis.

### 3.1. Clinical

Clinical results included socio-demographic/family history and the results of the different clinical scales for clinical assessment of depression severity, anxiety severity, personality and traumatic and stressful life events.

#### 3.1.1. Socio-Demographic/Family History

No significant differences were found between the two groups in terms of main sociodemographic characteristics (Table 1); although, subjects with Primary MD had a higher education level in comparison with participants in the AI-MD group. Regarding medical comorbidities, a significant difference was found (*p* = 0.026) with more subjects from the AI-MD group (54.5%) reporting this condition in comparison to Primary MD participants (29.8%). The majority of comorbidities included any hepatic disease and lipid metabolism disorders.

There were no significant differences with respect to hospitalization due to medical comorbidities. Most of the participants reported to be in pharmacological treatment with antidepressants without differences between Primary MD and AI-MD group (100% vs. 96.7% respectively; *p* = 0.37). Almost 80% of Primary MD participants provided information on history of depression in family members with differences between groups (*p* = 0.042). In contrast, a higher percentage of AI-MD participants reported alcohol and substance use disorders in their family history. Fifty-three percent of AI-MD patients and 28.3% of Primary MD group of patients reported a family history of alcohol use (*p* = 0.033). Finally, differences were also found for a family history of other substance use disorders (31.3% of AI-MD vs. 8.7% Primary MD, *p* = 0.016).

#### 3.1.2. Clinical Assessment

Characteristics of AUD in the AI-MD group were collected with a PRISM interview: The mean age of onset of alcohol abuse was 33.42 years (12.26 SD) and 37.34 years (12.49 DS) for alcohol dependence. According to the DSM-IV TR diagnosis criteria, 100% of subjects fulfilled a lifetime criteria for alcohol dependence and 94% for the last 12 months. The mean age for first alcohol disorder treatment in the AI-MD patients was 37.55 years (15.96 SD).

The main results in clinical severity for depression are described in Table 2. There were no differences in the age of onset of depression between the two groups. Moreover, there were no differences between groups for any of the instruments assessing the severity of depression (HAM-D, BDI and SSI) or anxiety (HAM-A and STAI). Furthermore, the severity of depression was not associated with the age of onset of alcohol addiction in the AI-MD group. A trauma and life events instrument (LSC-R) showed a higher mean score in patients with AI-MD diagnosis compared to Primary MD patients, ((14.21 ± 11.35 SD) vs. 9.30 ± 7.38 SD; *p* = 0.021)). There were no differences for the temperament and character (dimensions between groups). There were differences between groups regarding the following subscales: “disorderliness” trait from “novelty seeking” dimension (*p* = 0.035), showing AI-MD patients with higher scores in comparison to Primary MD patients (51.81 ± 10.05 and 46.29 ± 11.44, respectively). In addition, differences (*p* = 0.034) were found between groups regarding “conformity” trait from “reward dependence” dimension; patients in the Primary MD group showed a higher mean score (50.79 ± 9.41) than patients with an AI-MD diagnosis (44.87 ± 13.92).

Taking into account the DSM-IV-TR depression criteria for the diagnosis of depression, five (or more) criteria should be present during the same 2-week period and should represent a change from previous functioning; at least one of the symptoms is either: depressed mood or loss of interest or pleasure. The nine criteria are described in Table 2. First, there were no differences between groups for the first criteria (depressed mood), showing both groups with a similar prevalence of these criteria (97.9% in Primary MD vs. 94.1% in AI-MD). However, differences were found for the second criteria (anhedonia). The majority of Primary MD participants (97.9%) reported this symptom while a lower number in AI-MD participants reported it (82.4%) (*p* = 0.014). There were differences in relation to the third criterion, changes in weight and/or appetite; AI-MD patients showed a higher prevalence (91.2%) than patients with primary episodes (72.3%) (*p* = 0.036). There were also differences in the eight criteria (diminished ability to concentrate), more frequent among PMD patients (97.9%) than AI-MD group of patients (79.4%) (*p* = 0.006). Finally, differences were found in recurrent thoughts of death (criteria 9), where 63.8% of PMD patients showed these criteria and 29.4% of AI-MD patients (*p* = 0.002). There were no differences in other depression criteria.

### 3.2. Blood Test Results

Regarding the blood test results, AI-MD participants had more significant abnormal results in comparison with Primary MD in the following: TSH (*p* = 0.016), AST (*p* < 0.001), ALT (*p* < 0.001), ALP (*p* = 0.043) and GGT (*p* < 0.001). There were no significant differences in the results of CRP levels, bilirubin, cholesterol and triglycerides between groups. Table 3 shows the total number of participants (or percentage) with pathological results in both groups.

### 3.3. GWAS Results

**Figure 1:**
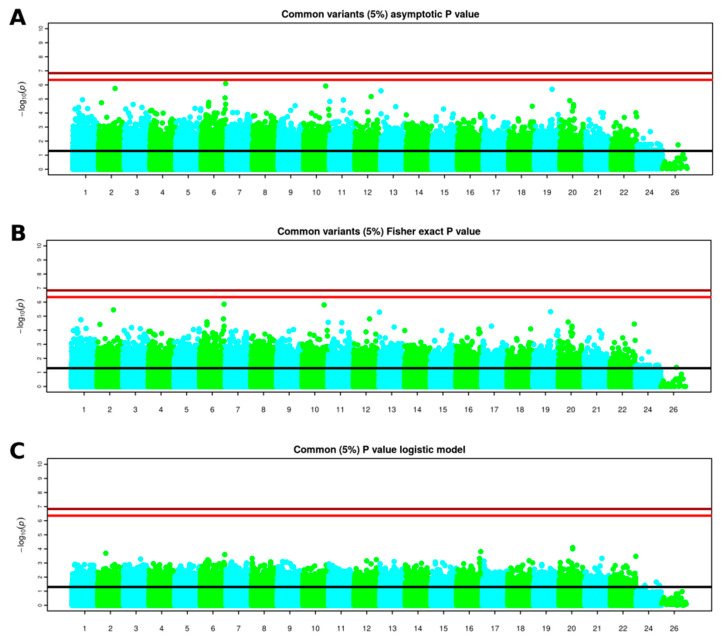
Manhattan plots indicating the negative base 10 logarithm of the *p*-values obtained performing: basic allele chi-square test (**A**) and Fisher’s exact test (**B**) and logistic regression model (**C**) on 341,946 common variants obtained from whole genome genotyping data. The black horizontal line represents a significance level of 0.05. The light red horizontal line represents the multiple testing obtained considering the number of independent loci; the dark red horizontal line represents the multiple testing threshold obtained considering the total number of considered common variants. Chromosomes over 22 represent sexual and mitochondrial chromosomes.

Variants with a missing rate higher than 5% or having a minor allele frequency lower than 1% or deviating significantly from Hardy–Weinberg equilibrium were filtered out. From the original 814,923 variants, 508,097 were considered for further analysis. A total of 24 samples were removed after quality control. For 16 individuals, unusual Identity By Descent (IBD) values were observed when compared to the rest of patients and were discarded due to possible contamination. An additional individual showed an heterozygosity rate deviating from the heterozygosity observed in the rest of patients. Principal component analysis (PCA) was performed to assess ancestry and seven patients with non-European ancestry were discarded for further analysis. As association analyses are generally performed considering only variants with a high frequency in the population, variants with a frequency lower than 5% in the sequenced samples were filtered out. For each single variant, among the 341,946 common variants for genotyping data, three different tests were performed separately: (i) basic allelic chi-square, (ii) Fisher’s exact test and (iii) logistic regression. A Manhattan plot resulted from each test (Figure 1). For each test, we created a table that contains the odd-ratios obtained (effect of the variants) and *p*-values, for more information about this tables see Table A1, Table A2 and Table A3.

**Figure 2:**
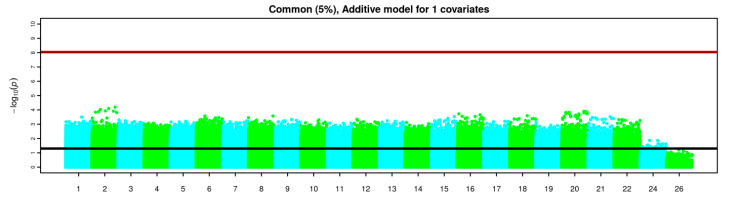
Manhattan plot indicating the negative base 10 logarithm of the *p*-values obtained when including each single covariate into the logistic regression model for 341,946 common variants from whole genome genotyping data. For each individual variant, a single test for each covariate was performed, so for each variant, 16 different tests were performed and each test is represented by a point in the Manhattan plot. The black horizontal line represents a significance level of 0.05. The dark red horizontal line represents the multiple testing threshold obtained considering the total number of performed tests (Number of variants × Number of covariates). Chromosomes over 22 represent sexual and mitochondrial chromosomes.

Apart from testing each single variant, covariates were also included into the logistic model. When including all the provided covariates into the logistic model even the most minimal differences between two groups of samples disappeared, suggesting that correcting for all these covariates jointly is not useful to identify the genetic differences between the two groups of individuals in our study. On the other hand, covariates were also analysed separately in Figure 2 and none of the variants reached statistical significance.

Overall, none of the variants reached significance beyond multiple testing thresholds, although, some suggestive variants were observed in chromosomes 2, 6, 10, 13 and 19 in the basic allele chi-square test and Fisher’s exact test (Figure 2).

Interestingly, variants rs3130531, rs7772901, rs73115241, rs386580033 and rs529060937 were among the top 20 variants for all the three different applied association tests; moreover 17 over the 20 SNPs listed in Table 4 were also represented in Table 4 meaning that a basic allele chi-square test and Fisher’s exact test produced very similar results. Covariates analyses in a regression model did not provided any significant result. Table 4 shows further information of the five relevant variants.

## 4. Discussion

AI-MD is a common and clinically relevant condition that should be better characterized to improve its diagnosis and adequate treatment. This study has found clinical and biological features that may help physicians in differentiating AI-MD from primary MD and improve the knowledge about their etiopathology and also, its therapeutic approach. Clinical differences were found mainly in family history of diseases; criteria used for depression diagnosis, lifetime traumatic stressors and medical comorbidities. However, non-genetic differences were found.

AI-MD patients showed greater alcohol use and a family history of other substances use disorders, whilst in contrast, MD patients showed greater family history of depression. Interestingly, AI-MD patients showed greater lifetime stressors events such as physical abuse, childhood abuse, intimate partner violence, etc. These findings are consistent with other animal and human studies reporting an association between traumatic events and SUDs [63,64,65,66,67]. Furthermore, as expected, AI-MD showed more medical comorbidity possibly by the effects related to the alcohol use, and its toxicity [68]. Finally, overall, personality dimensions and traits did not show large differences between groups.

We identified some further differences in the criteria used to diagnose MD according to DSM-IV-TR [69]. We only found differences in four of the nine criterions used to diagnose depression. AI-MD patients met with more frequency the criteria related to changes in weight. The high medical comorbidity found among AI-MD patients may explain this significant difference related to weight criteria [70,71] although we have not detected this association in our sample. Other authors have found different criteria between Primary and Induced depression associated with cocaine use disorder [72]; however, these authors found more “weight changes” in the primary depression group compared to induced depression group. In contrast, Primary MD patients met more criteria related to anhedonia, loss of concentration and recurrent thoughts of death. Our results are not according to other studies and show that depressive co-morbidity in patients with AUD may thus be characterized by more pronounced levels of anhedonia, as compared to other symptom domains of depression [73]. Animal and human studies were the focus in the paper of anhedonia as a transdiagnostic symptom. Anhedonia is a core symptom in depression and it is also involved in addictive disorders [74]. In this sense, dysregulation of the reward system and alterations in ventral extrapyramidal circuits were described in both disorders [75,76]. These findings imply acute dysfunction within mesolimbic dopamine pathways, although the cause of such alterations is unclear [77].

The differences in the blood test in terms of liver enzymes having a greater prevalence of abnormal results in the AI-MD group, in the same line as the severe medical condition, were expected due to the well recognized association between alcohol use and liver disease (for review Fuster and Samet, 2018) [78], ranging from steatosis to cirrhosis and liver cancer. A relationship between liver disease, AUD and depressive symptoms has also been described [79]; the underlying mechanism could be associated with inflammatory processes that are worsened by alcohol consumption [80]. Finally, animal and human studies have described an association between changes in the hypothalamus–pituitary–thyroid (HPT) axis and AUD [81]; these changes seems to normalize after detoxification [82,83]. The mechanism that has been related with changes in TSH levels is that alcohol could affect the feedback inhibition of the thyroid hormones by having a direct toxic effect on the thyroid gland and a compensatory increase in the thyroid release hormone secretion.

Regarding GWAS findings, single variant association analysis did not produce any significant result nor when including clinical covariates (jointly, separately or combinations of them). Nevertheless, some suggestive variants were identified: 5 SNPs having the lowest P-values for the 3 types of statistical analysis were: rs3130531, rs7772901, rs73115241, rs386580033 and rs529060937. As far as we know, none of those SNPs have been previously associated to depression, nor alcohol use disorder. For the rs3130531, the T allele was more prevalent in the AI-MD group compared to Primary MD group of patients. This SNP has been implicated in somatic illness as rheumatoid arthritis [84] and diabetes [85], but at this moment, no association has been described previously with depression nor AUD. The rs73115241 is an intergenic variant, located in Chromosome 20 with no currently known function. The T allele was more prevalent in the AI-MD group compared to the Primary MD group. The rs7772901, is an intronic variant; in our sample, the C allele was more prevalent in the AI-MD group than in the Primary MD group of patients. Finally, rs386580033 and rs529060937 correspond both to intronic variants, probably with a regulatory function. In our sample, the A and the G allele, respectively, were more prevalent in the Primary MD group than in the AI-MD diagnosed patients.

Our findings have some limitations that should be considered. The main limitation is related to the small sample size and not having control groups to compare (healthy controls and AUD non-depressed controls). The analysis performed did not show differences in women, but this could be related with the sample size, which has made it not possible to study the effect of gender. Depressive disorders are more common in women than men, moreover, depression associated with addictive disorders (either primary or induced) is more prevalent in women with SUD than in men, and more frequent than expected in women without any SUD [86]. Differences have also been found in clinical presentation and some neurobiological markers [87]. A bigger sample size could help to detect gender differences. Furthermore, replication is required in an independent set of samples and/or using alternative and more complex genomic risk score methods. In addition, MD and AUD has a modest heritability, both are polygenic disorders meaning that many genetic variants have an individual small effect size. Finally, due to the effects of alcohol consumption in inflammatory pathways which also have been related with depression, it would be important to replicate these findings, comparing them with a group of AUD without any depression.

In spite of these limitations, the accurate process of phenotype and genotype of the samples is a strong point of the study. AI-MD has crucial implications for both prognosis and therapeutic approaches. In two previous meta-analyses of antidepressant treatments in comorbid depression with substance use, the lack of response to selective serotonin reuptake inhibitors (SSRIs) was explained by the possible confounding factor of the presence of substance-induced depression in the samples [17,18]. In this context, the distinction between Primary MD and AI-MD might be crucial to improve treatment strategies and outcomes. To date, the diagnosis is based on clinical criteria (using DSM-5 (American Psychiatric Association 2013) or ICD-10 (Organización Mundial de la Salud 2000)) but there is still a need for specific biomarkers to facilitate the identification of AI-MD to improve diagnosis and clinical management. In this sense, genetic studies including expression studies, pharmacogenomics and epigenetics can improve the diagnosis, therapeutic approach and prognosis of these prevalent diseases.

## 5. Conclusions

This preliminary study has found clinical and biological features that may help physicians in differentiating AI-MD from primary MD. These results will facilitate future studies to increase the knowledge about their etiopathology and its therapeutic approach.

## Figures and Tables

**Table 1:** Sociodemographic and family history data.

Sample Characteristics	Primary MD	AI-MD	*p* ^a^
	N = 47 (%)	N = 33 (%)	
Age (Mean ± SD)	49.87 ± 11.32	50.39 ± 8.89	0.140 ^b^
Gender			0.678
Men	22 (46.8)	17 (51.5)	
Women	25 (53.2)	16 (48.5)	
Household structure			0.736
Alone	14 (29.8)	11(33.3)	
With others	33 (70.2)	22(66.7)	
Education level			0.041
Primary or Secondary education	20 (42.6)	21 (65.6)	
Upper secondary education	27(57.4)	11 (34.4)	
Employment situation			0.271
Employed	16 (34)	6 (18.8)	
Unemployed	1 (2.1)	0	
Disability	27 (57.4)	25 (78.1)	
Retired	3 (6.4)	1 (3.1)	
Medical comorbidities			
Serious illness (SI)	14 (29.8)	18 (54.5)	0.026 *
Hospitalization due to SI ^c^	14 (100)	17 (94.4)	0.370
Current medication ^d^	46 (100)	29 (96.7)	0.213
Family History			
Depression ^e^	35(79.5)	17(56.7)	0.042 *
Alcohol use disorder ^f^	13 (28.3)	16(53.3)	0.033 *
Substance use disorder ^g^	4 (8.7)	10(31.3)	0.016 *

Notes: ^a^ Chi-Square; ^b^ Student’s T-Test; ^c^ n = 32; ^d^ n = 76; ^e^ n = 74; ^f^ n = 76; ^g^ n = 78; * Significance (*p* < 0.05). MD: Major Depression; AI-MD: Alcohol Induced Major Depression.

**Table 2:** Results of Clinical Assessment on depression, anxiety, personality and stressful events.

Variables	Primary MD	AI-MD	*p* ^a^
	N = 47 (Mean ± SD/Mean (%))	N = 33 (Mean ± SD/Mean (%))	
Age onset depression (years)	37.64 (13.53)	39.18 (11.26)	0.593
HAM-D	15.64 ± 10.34	11.88 ± 7.54	0.79
BDI	22.37 ± 14.65	23.41 ± 11.59	0.739
SSI	11.68 ± 8.12	12.36 ± 8.48	0.156
HAM-A	25.22 ± 14.32	25.67 ± 12	0.884
STAI			
STAI- State	28.17± 13.82	27.44 ± 13.78	0.817
STAI- Trait	30.00 ± 13.16	32.28 ± 11.17	0.425
LSC-R	9.30 (7.38)	14.21 (11.35)	0.021 *
Personality Dimensions			
*Temperament*			
Novelty seeking (NS)	47.38± 11.07	50.84 ± 9.89	0.172
Harm avoidance (HA)	54.60 ± 11.82	60.87 ± 11.61	0.415
Reward dependence (RD)	43.57 ± 9.65	45.68 ± 10.66	0.381
Persistence (PS)	44.45 ± 9.92	47.55 ± 11.62	0.224
*Character*			
Self-directedness (SD)	42.33 ± 11.92	39.61 ± 11.12	0.325
Cooperativeness (CO)	45.14 ± 11.42	45 ± 12.22	0.959
Self-transcendence (ST)	48.74 ± 10.57	50.35 ± 11.53	0.536
Depression Criteria			
Criteria 1: depressed mood	46 (97.9)	32 (94.1)	0.377
Criteria 2: diminished interest or pleasure	46 (97.9)	28 (82.4)	0.014 *
Criteria 3: significant unintentional weight loss or gain	34 (72.3)	31 (91.2)	0.036 *
Criteria 4: insomnia or sleeping too much	43 (91.5)	27 (79.4)	0.117
Criteria 5: agitation or psychomotor retardation	34 (72.3)	22 (64.7)	0.463
Criteria 6: fatigue	44 (93.6)	27 (79.4)	0.055
Criteria 7 feelings of worthlessness or excessive guilt	43 (91.5)	28 (82.4)	0.217
Criteria 8: diminished ability to think or concentrate	46 (97.9)	27 (79.4)	0.006 *
Criteria 9: recurrent thoughts of death	30 (63.8)	10 (29.4)	0.002 *

Notes: ^a^ Student’s T-Test * Significance (*p* < 0.05). HAM-D: Hamilton Depression Rating Scale, BDI: Beck Depression Inventory, SSI: Suicidal Ideation Scale, HAM-A: Hamilton Anxiety Rating Scale, STAI: State-Trait Anxiety Inventory, LSC-R: Life Stressor Checklist-Revised.

**Table 3:** Results of pathological blood test in Primary MD and AI-MD groups.

Biochemical Paramaters	Subjects with Abnormal Values *		*p*
	Primary MD	AI-MD	
(Normal Values)	N (%)	N (%)	
TSH ^a^ (10–38 mcUI/mL)	0	4 (12.5)	0.016 **
Bilirubin ^b^ (0.2–1.2 mg/dL)	2 (5)	4 (12.1)	0.270
AST ^c^ (UI/L) 10–38 UI/L	4 (9.3)	17 (51.5)	<0.001 **
ALT ^d^ (UI/L) 7–41 UI/L	14 (32.6)	26 (76.5)	<0.001 **
ALP ^e^ (40–129 UI/L)	3 (8.6)	9 (27.3)	0.043 **
GGT ^f^ (8–61 UI/L)	11 (32.4)	26 (78.8)	<0.001 **
Cholesterol ^g^ (50–129 mg/dL)	25 (59.5)	16 (48.5)	0.340
Triglycerides ^h^ (40–150 mg/dL)	14 (33.3)	7 (21.9)	0.279
CRP ^i^ (0–0.8 mg/dL)	13 (31.7)	15 (57.7)	0.378

Notes: ^a^ Chi-Square. * There were no patients with values below the lower range in all the parameters analysed. The parameters were considered abnormal when the value was above the highest range; ** Significance (*p* < 0.05), Thyroid-stimulating hormone (TSH), alanine transaminase (ALT), aspartate transaminase (AST), alkaline phosphatase (ALP), and gamma-glutamyl transpeptidase (GGT), C Reactive Protein (CRP). ^a^ n = 76, ^b^ n = 73, ^c^ n = 76, ^d^ n = 77, ^e^ n = 68, ^f^ n = 67, ^g^ n = 74, ^h^ n = 74, ^i^ n = 67.

**Table 4:** Genetic information of the five relevant variants.

SNP	Gene	Function	Probeset ID	Genotype Category
rs3130531		intergenic	AX-11435435	PolyHighResolution
rs7772901	PDE10A	intron variant	AX-11644567	PolyHighResolution
rs73115241		intergenic	AX-13511810	PolyHighResolution
rs386580033	PSORS1C1	intron variant	AX-35729741	PolyHighResolution
rs529060937	PSORS1C1	intron variant	AX-35729743	PolyHighResolution

## Appendix A

For the purpose of this report, a table reporting those relevant differences (not achieving statistically significance) in allele frequency between the two groups were created for each test. The tables only contain those variants showing higher differences between groups. For each table we report different fields (columns) according to the specific test conducted. The psychical position (Pos) reported in the tables refers to GRCH 38 version from the Genome Reference Consortium. In Table A1, Table A2 and Table A3 are listed the 20 SNPs showing the lowest *p*-values for basic allele chi-square test, Fisher’s exact test, and logistic regression, respectively.

**Table A1:** Basic allele chi-square test.

SNP	Chr	Pos	Effect Allele	Alternative Allele	F_AI-MD	F_Primary MD	OR	*p* Value
rs73250026	6	165960669	G	A	0.35	0.01429	37.15	7.991 × 10^−7^
rs12355672	10	123921288	A	G	0.3	0	NA	0.000001204
rs2602186	2	159271306	A	G	0.3421	0.01471	34.84	0.000001802
rs2245046	19	47858424	A	G	0.3684	0.02857	19.83	0.000002057
rs61955462	13	21009654	A	G	0.45	0.07143	10.64	0.000002643
rs76785029	12	94882905	T	C	0.3421	0.02857	17.68	0.000006816
rs77332950	6	162137147	T	C	0.375	0.04412	13	0.000008363
rs11163044	1	81002495	T	C	0.25	0	NA	0.00001147
rs61893521	11	76392642	A	G	0.425	0.07353	9.313	0.0000119
rs73124405	20	20515790	T	G	0.3421	0.0303	16.64	0.00001311
rs10839772	11	1850324	A	G	0.55	0.1571	6.556	0.00001524
rs3130531	6	31206616	A	G	0.7105	0.2794	6.33	0.00001749
rs116179105	2	19494199	A	G	0.2895	0.01471	27.3	0.00001855
rs7772901	6	165959846	C	A	0.475	0.1143	7.012	0.00002349
rs28504201	3	58573163	A	G	0.4	0.07143	8.667	0.00002465
rs73115241	20	38797004	T	C	0.425	0.08571	7.884	0.00002561
rs386580033	6	31091163	A	G	0.2	0.6176	0.1548	0.00002629
rs2771040	9	108152199	G	A	0.4737	0.1143	6.975	0.00003021
rs529060937	6	31091197	G	A	0.2105	0.6286	0.1576	0.00003293
rs73485007	18	74495070	T	C	0.2778	0.01471	25.77	0.00003323

Notes: Chr: Chromosome, SNP: SNP ID, Pos: Physical position (base-pair), F_AI_MD: Frequency of this allele in AI-MD, F_Primary MD: Frequency of this allele in Primary MD, OR: Estimated odds ratio, P: Asymptotic *p*-value for this test.

**Table A2:** Fisher’s exact test.

SNP	Chr	Pos	Effect Allele	Alternative Allele	F_AI-MD	F_Primary MD	OR	*p* Value
rs73250026	6	165960669	G	A	0.35	0.01429	37.15	0.000001416
rs12355672	10	123921288	A	G	0.3	0	NA	0.000001588
rs2602186	2	159271306	A	G	0.3421	0.01471	34.84	0.000003575
rs2245046	19	47858424	A	G	0.3684	0.02857	19.83	0.000004805
rs61955462	13	21009654	A	G	0.45	0.07143	10.64	0.000005148
rs77332950	6	162137147	T	C	0.375	0.04412	13	0.00001555
rs76785029	12	94882905	T	C	0.3421	0.02857	17.68	0.00001572
rs11163044	1	81002495	T	C	0.25	0	NA	0.00001807
rs386580033	6	31091163	A	G	0.2	0.6176	0.1548	0.00002548
rs73124405	20	20515790	T	G	0.3421	0.0303	16.64	0.00002572
rs10839772	11	1850324	A	G	0.55	0.1571	6.556	0.00002733
rs61893521	11	76392642	A	G	0.425	0.07353	9.313	0.0000293
rs3130531	6	31206616	A	G	0.7105	0.2794	6.33	0.00003134
rs137916	22	50491713	A	G	0.025	0.3529	0.04701	0.00003637
rs116179105	2	19494199	A	G	0.2895	0.01471	27.3	0.00003841
rs529060937	6	31091197	G	A	0.2105	0.6286	0.1576	0.00004234
rs7772901	6	165959846	C	A	0.475	0.1143	7.012	0.00005108
rs915476	17	32288009	C	T	0	0.2857	0	0.00005113
rs73115241	20	38797004	T	C	0.425	0.08571	7.884	0.00005121
rs17780066	13	78448090	T	C	0.2368	0	NA	0.00005933

Notes: Chr: Chromosome, SNP: SNP ID, Pos: Physical position (base-pair), F_AI_MD: Frequency of this allele in AI-MD, F_Primary MD: Frequency of this allele in Primary MD, OR: Estimated odds ratio, P: Asymptotic *p*-value for this test.

**Table A3:** Logistic regression.

SNP	Chr	Pos	Effect Allele	OR	*p* Value
rs73115241	20	38797004	T	14.1	0.00008067
rs6028915	20	38786218	C	13.63	0.0001002
rs9933149	16	87226206	T	0.06377	0.0001541
rs2162380	2	64556555	A	12	0.0002039
rs7772901	6	165959846	C	9.49	0.0002534
rs2301584	22	51171497	A	11.89	0.0003368
rs4876226	8	2059004	T	9.259	0.0004704
rs4876226	8	2059004	T	9.259	0.0004704
rs4876226	8	2059004	T	9.259	0.0004704
rs16843122	3	135278749	C	0.08456	0.0005255
rs4765145	12	124843104	C	0.05933	0.0005727
rs3130531	6	31206616	A	5.473	0.0005918
rs386580033	6	31091163	A	0.1706	0.0006138
rs7407243	18	70010868	G	9.425	0.000684
rs529060937	6	31091197	G	0.1759	0.0007074
rs4913427	12	68631620	T	0.1561	0.0007113
rs499691	6	32194339	T	7.06	0.0007266
rs1048677	17	3564716	G	7.575	0.0007284
rs6046396	20	19852503	G	6.717	0.0007339
rs34058147	13	75567543	G	0.1383	0.0007866

Notes: Chr: Chromosome, SNP: SNP ID, Pos: Physical position (base-pair), F_AI_MD: Frequency of this allele in AI-MD, F_Primary MD: Frequency of this allele in Primary MD, OR: Estimated odds ratio, P: Asymptotic *p*-value for this test.
